# Selective Diffusive Gradients in Thin Films (DGT) for the Simultaneous Assessment of Labile Sr and Pb Concentrations and Isotope Ratios in Soils

**DOI:** 10.1021/acs.analchem.2c00546

**Published:** 2022-04-15

**Authors:** Stefan Wagner, Jakob Santner, Johanna Irrgeher, Markus Puschenreiter, Steffen Happel, Thomas Prohaska

**Affiliations:** † Department General, Analytical and Physical Chemistry, Chair of General and Analytical Chemistry, Montanuniversität Leoben, Franz-Josef-Strasse 18, 8700 Leoben, Austria; ‡ Department of Chemistry, Institute of Analytical Chemistry, University of Natural Resources and Life Sciences, Vienna, Konrad-Lorenz-Strasse 24, 3430 Tulln, Austria; § Department of Crop Sciences, Institute of Agronomy, University of Natural Resources and Life Sciences, Vienna, Konrad-Lorenz-Strasse 24, 3430 Tulln, Austria; ∥ Department of Forest and Soil Sciences, Institute of Soil Research, University of Natural Resources and Life Sciences, Vienna, Konrad-Lorenz-Strasse 24, 3430 Tulln, Austria; ⊥ TrisKem International, 3 Rue des Champs Géons, ZAC de l’Eperon, 35170 Bruz, France

## Abstract

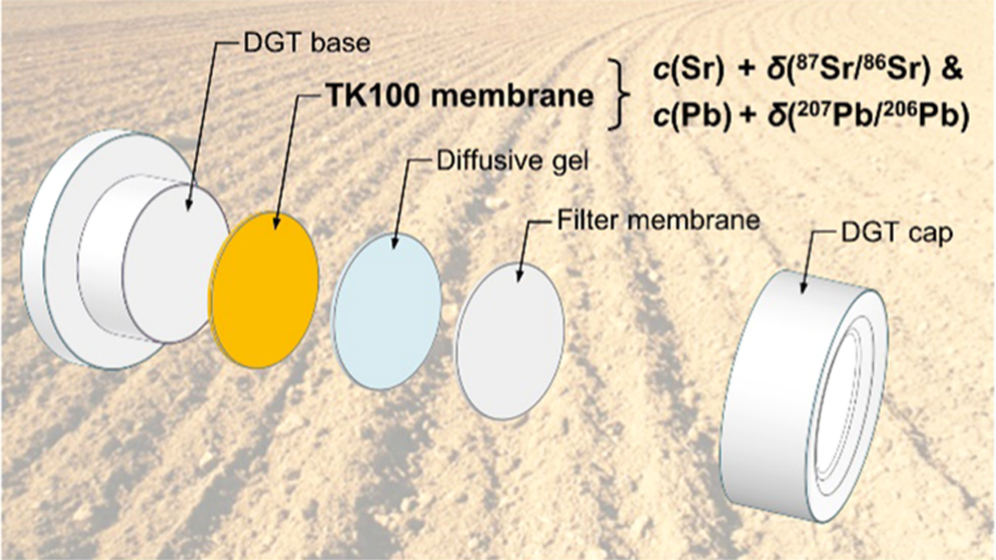

A method using diffusive gradients in thin films (DGT) for the accurate quantification of trace-level (μg L^–1^) Sr and Pb concentrations and isotope ratios [*δ*_SRM 987_(^87^Sr/^86^Sr) and *δ*_SRM 981_(^207^Pb/^206^Pb)] in labile, bioavailable element fractions in soils is reported. The method is based on a novel poly(tetrafluoroethylene) (PTFE) membrane binding layer with combined di(2-ethyl-hexyl)phosphoric acid (HDEHP) and 4,4′(5′)-bis-*t*-butylcyclohexano-18-crown-6 (crown-ether) functionality with high selectivity for Sr and Pb (TK100 membrane). Laboratory evaluation of the TK100 DGT showed linear uptake of Sr over time (2–24 h) up to very high Sr mass loadings on TK100 membranes (288 μg cm^–2^) and effective performance in the range of pH (3.9–8.2), ionic strength (0.001–0.1 mol L^–1^), and cation competition (50–160 mg L^–1^ Ca in a synthetic soil solution matrix) of environmental interest. Selective three-step elution of TK100 membranes using hydrochloric acid allowed us to obtain purified Sr and Pb fractions with adequate (≥75%) recovery and quantitative (≥96%) matrix reduction. Neither DGT-based sampling itself nor selective elution or mass loading effects caused significant isotopic fractionation. Application of TK100 DGT in natural soils and comparison with conventional approaches of bioavailability assessment demonstrated the method’s unique capability to obtain information on Sr and Pb resupply dynamics and isotopic variations with low combined uncertainty within a single sampling step.

Strontium (Sr) and lead (Pb) are ubiquitous trace elements in the environment whose variations in elemental content and isotopic composition in bioavailable soil fractions are widely used for both tracing and understanding biogeochemical cycles in nature.^1−3^ An accurate assessment of soil Sr and Pb bioavailability is thus crucial in many fields of research, as highlighted by recent applications in archeology, geology, paleoecology, (food) forensics, and environmental science.^4−9^

In the past, numerous methods have been proposed to assess the bioavailable fractions of Sr and Pb in soil. Most commonly, soil solution sampling or equilibrium-type chemical soil extractions using nonbuffered salt solutions (*e.g.*, NH_4_NO_3_, *c* = 1 mol L^–1^) are applied.^10−12^ However, these extracts exhibit complex and variable compositions with high matrix loads, complicating accurate Sr and Pb measurements by inductively coupled plasma mass spectrometry (ICP-MS). As a result, laborious sample purification strategies are required. The diffusive gradients in thin films (DGT) technique^23^ provides an alternative sampling strategy, offering the potential to assess Sr and Pb concentrations and isotope ratios in labile, bioavailable fractions in soils accurately and cost-effectively. DGT is an infinite-sink technique using hydrogel layers containing binding agents (*e.g.*, ion-exchange resins, oxides) with high selectivity for the target analyte(s). Applied in soil, DGT samples kinetically labile analyte fractions, consisting of free ions, complexes with inorganic and organic ligands, and exchangeable solid phase pools,^13−15^ which usually comprise the bioavailable fraction. Consequently, DGT has been shown to be a better predictor for nutrient and contaminant uptake by plants under diffusion-limited conditions while performing equally well to chemical extractants in nondiffusion-limited cases.^15^ Moreover, the unique capability of DGT for analyte preconcentration and *in situ* matrix separation may substantially facilitate sample preparation for multicollector (MC) ICP-MS, as recently shown for DGT-based sulfur stable isotope measurements in labile soil sulfate.^16^

Yet, the analysis of Sr by DGT represents unique challenges, as evidenced by the lack of an adequate DGT method for sampling Sr. Until now, only two research groups have trialed to assess Sr by DGT quantitatively. The first study on Sr uptake from aqueous solutions by DGT was reported by Chang et al.,^17^ who used a general cation-exchange resin (AG50W-X8) as the binding agent. The study showed that DGT-measured Sr concentrations agreed well with those in natural freshwater. However, lacking selectivity for Sr, the binding layer was quickly saturated by codissolved cations, limiting its application in DGT to short deployment times (≤20 h) and soft water environments with a very low ionic background (≤0.001 mol L^–1^). In the second study, Garmo et al.^18^ tested Chelex DGT for the measurement of Sr among 54 other elements under laboratory conditions. While this technique works well for divalent transition metals and can also be used for isotope ratio measurements of Pb in aquatic systems,^19^ its performance for sampling Sr is affected by the low binding affinity of the Chelex resin for alkaline earth metals, causing pH-dependent H^+^ competition effects.^18^

In this study, we take advantage of the selective adsorption of Sr by a new binding phase with combined di(2-ethyl-hexyl)phosphoric acid (HDEHP) and 4,4′(5′)-bis-*t*-butylcyclohexano-18-crown-6 (crown-ether) functionality (TK100). The HDEHP binds Sr^2+^ on an organic stationary phase *via* a H^+^ ion-exchange mechanism, thereby facilitating complexation of Sr by the crown-ether. Surman et al.^20^ investigated TK100 in the resin form for batch- and column-type separations of ^90^Sr from interfering radionuclides and confirmed that it selectively adsorbed Sr from natural water samples with quantitative recovery (93% in HCl, *c* = 2 mol L^–1^) under environmentally relevant conditions (pH 2–8; ionic strength 0–0.5 mol L^–1^). Additionally, they demonstrated that TK100 shows a particularly high affinity for Pb, indicating the possibility for adsorptive cosampling of both Sr and Pb from the same sample. Therefore, we evaluated TK100 as a selective binding phase for the simultaneous measurement of labile Sr and Pb concentrations under natural competitive conditions by DGT for the first time. Moreover, we investigated if TK100 DGT allows for direct matrix separation without significant isotopic fractionation during solute sampling to enable accurate determination of Sr and Pb isotope ratios by MC ICP-MS. The new method was comprehensively evaluated under laboratory conditions and tested on different types of natural soil samples.

## Experimental Section

Details on laboratory procedures and materials are provided in the Supporting Information. To comply with notation conventions used in the DGT literature, the symbol *c* is used throughout this work for both molar and mass concentrations in solution instead of the designated symbols *c* and γ recommended by the International Union of Pure and Applied Chemistry (IUPAC).

### Mass Spectrometric Analyses

Elemental mass concentrations in sample solutions were measured using quadrupole single-collector ICP-MS (Elan 9000 DRCe, Perkin Elmer). External calibration and internal normalization using indium were applied as reported previously.^21^ In-house quality control standards (prepared by mixing certified multi- and single-element standards) reflecting sample compositions were analyzed every 6–12 samples for quality assurance. Analytical recoveries were typically within ±5% of the target value. Isotope ratio measurements were accomplished using Nu Sapphire (SP017) and Nu Plasma HR (NP048) sector-field MC ICP-MS instruments (both Nu Instruments, U.K.). Sample and standard solutions were diluted to obtain Sr and Pb mass concentrations of 20–75 and 2–15 μg L^–1^, respectively. Standard-sample bracketing (SBB) was applied using solutions of SRM 987 (SrCO_3_; NIST) for Sr and SRM 981 for Pb (≥99.9% purity lead metal; NIST) as the isotopic reference.^21^ Sample and standard solutions of Sr were spiked with Zr to enable internal interelemental correction of the instrumental isotopic fractionation (IIF). Pb isotope ratios were corrected for IIF by SSB. Outlier removal, blank correction, and correction of residual Rb (in Sr measurement solutions only) were accomplished as reported previously.^22^ Details of instrumental setups and parameters of the MC ICP-MS analyses of Sr and Pb for different experiments are provided in Table S1. All samples were measured in diluted HCl (*w* = 2%).

### TK100 Membrane Preparation

We aimed to develop a TK100-containing DGT binding layer using polyacrylamide- or polyurethane-based hydrogel matrices typically used in DGT applications. Despite our efforts to improve the gel formulation, initial tests showed that the Sr binding functionality of the TK100 resin (particle size 100–150 μm; TrisKem International, FR) is compromised when incorporated into these hydrogel matrices (Figure S1). To overcome this problem, a poly(tetrafluoroethylene) (PTFE) membrane (6 cm diameter, 0.7 mm thickness), which had not been used for DGT binding layers before, was used as the reversed-phase support for the TK100 extractant system. The PTFE membrane was impregnated with TK100 at a mass fraction of 37 ± 3% (TrisKem International). The TK100 membranes were cut to discs (*A*_disc_ = 4.52 cm^2^) using a stainless-steel cutter, precleaned in HNO_3_ (*c* = 0.5 mol L^–1^) for 6 h under constant shaking, thoroughly rinsed with water until the pH of the storage water was ∼5, and then stored in water at 6 °C in the dark until use.

### Assembly of DGT Devices

TK100 DGT devices were assembled by stacking a TK100 membrane disc, an agarose cross-linked polyacrylamide (APA) diffusive gel (0.8 mm thickness),^23^ and a poly(ether sulfone) (PES) filter membrane (0.15 mm thickness, 0.45 μm pore size; Sartorius, DE) on top of each other into DGT moldings. APA gels and PES membranes were pre-equilibrated in NaNO_3_ (*c* = 0.01 mol L^–1^) for ≥24 h to avoid charge-induced disequilibrium artifacts.^17^ Assembled DGT devices were stored at room temperature in plastic zip-lock bags containing ∼5 mL of NaNO_3_ (*c* = 0.01 mol L^–1^) until use.

### Calculation of DGT Concentrations and Isotope Ratios

The determination of the total analyte mass, *m*, bound by the TK100 membrane disc in the DGT device according to eq 1 allows one to calculate time-averaged concentrations of labile analyte species at the probe–medium (*i.e.*, solution or soil) interface, *c*_DGT_, using eq 2(23)
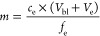
1
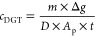
2where *c*_e_ (μg L^–1^) is the analyte mass concentration in the eluate, *V*_bl_ (mL) is the TK100 membrane disc volume, *V*_e_ (mL) is the eluent volume, *f*_e_ is the analyte elution factor, Δ*g* (cm) is the diffusive-layer thickness, *D* (cm^2^ s^–1^) is the diffusion coefficient of the analyte in the diffusive layer, *A*_p_ (cm^2^) is the sampling window area of the DGT device, and *t* (s) is the deployment time. Sr and Pb isotope ratios in sample (spl) solutions are reported relative to an isotopic reference standard (std; *i.e.*, SRM 987 for Sr and SRM 981 for Pb) using the delta (*δ* in ‰) notation according to eq 3. The possible isotopic fractionation by DGT was calculated as the difference between the mean *δ* values of the DGT eluate (DGT) and the reference solution (ref), *i.e.*, the immersion solution (soln) or the NH_4_NO_3_ soil extract (NH_4_NO_3_), using eq 4
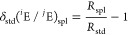
3
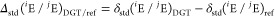
4where ^*i*^E is the heavier isotope *i* of the element E (*i.e.*, ^87^Sr or ^207^Pb), ^*j*^E is the lighter isotope *j* of the element E (*i.e.*, ^86^Sr or ^206^Pb), and *R* is the corresponding isotope ratio of the sample or standard after blank and interference correction. Details on the estimation of combined measurement uncertainties (*u*_c_) are provided in the Supporting Information. Significance of differences between two mean values was determined considering their expanded uncertainties (*U*, *k* = 2).^24^ Unless stated otherwise, all values in the text are reported as means ±*U* (*k* = 2).

### Analyte Uptake and Elution Recovery

The uptake efficiencies, *f*_u_, and elution recoveries, *f*_e_, of Sr and Pb by TK100 membrane discs were evaluated in mass balance experiments using ICP-MS analysis.^17^ The discs were deployed in quadruplicates for 24 h in 10 mL of well-shaken NaCl solution (*c* = 0.01 mol L^–1^) containing Sr and Pb (both *c* = 500 μg L^–1^) at pH 5.2. The discs were retrieved from the immersion solution using pipette tips and tweezers, rinsed two times on each side with water using a spray bottle, and placed into polyethylene (PE) vials. The elution recovery was determined by batch elution for 24 h in well-shaken 10 mL of HCl (*c* = 6 mol L^–1^), which was based on previous works on the TK100 resin, indicating minimal binding of both Sr and Pb under these conditions.^20,25,26^ Each experiment was repeated at least two times on different days to assess the uptake and recovery reproducibility.

### Analyte Selectivity and Chromatographic Separation

The selectivity of TK100 membranes was evaluated in an additional mass balance experiment using the same immersion conditions as stated above but further adding analytes, which interfere in subsequent MC ICP-MS analysis: Ca (*c* = 5000 μg L^–1^), Rb (*c* = 500 μg L^–1^), and Hg (*c* = 500 μg L^–1^). To test a potential three-step elution scheme to chromatographically separate Sr and Pb from the potentially cobound matrix interferences, the loaded TK100 discs were retrieved, rinsed, and placed into PE vials as described above. In the first step, elution for 1 h in 10 mL of either HNO_3_ (*c* = 8 mol L^–1^) or HCl (*c* = 0.01 mol L^–1^) was tested as a matrix wash to remove Ca, Rb, and Hg with minimal losses of Sr and Pb. In the second step, TK100 discs were placed in 10 mL of HCl (*c* = 2 mol L^–1^) for 24 h to elute Sr. In the third step, 10 mL of HCl (*c* = 6 mol L^–1^) for another 24 h was used to elute more strongly bound Pb. Constant shaking was applied in all elution steps using a horizontal shaker. Between each step, TK100 discs were rinsed two times on each side with water using a spray bottle to avoid carry-over. The elemental masses in each eluate fraction were determined after ICP-MS analysis and compared to those taken up by the TK100 discs to calculate the recovery in each fraction individually.

### Blanks, Detection Limits, and Quantification Limits

Blanks were assessed using TK100 DGT devices, which were treated as the experimental devices but not exposed to the deployment medium. Method detection limits (*MDL*s) and method quantification limits (*MQL*s) were calculated as three and ten times the standard deviation (*SD*) of the DGT blanks.

### Measurement of Diffusion Coefficients

A diaphragm diffusion cell was used to determine the diffusion coefficient, *D*, of Sr in APA diffusive gel.^17^ Details of the diffusion cell experiment are provided in the Supporting Information. For Pb, the conventional *D* value (8.03 × 10^–6^ cm^2^ s^–1^ at 25 °C) provided by DGT Research Ltd. was used.^27^ Diffusion coefficients were corrected for temperature using the Stokes–Einstein equation.^23^

### Effect of pH and Ionic Strength

The effect of pH and ionic strength was evaluated by deploying triplicate TK100 DGT devices for 24 h in 2 L of well-stirred solutions spiked with both Sr (*c* = 500 μg L^–1^) and Pb (*c* = 50 μg L^–1^) at varying pH values [3.9–8.2; *c*(NaNO_3_) = 0.01 mol L^–1^] or ionic strengths [*c*(NaNO_3_) = 0.001–0.75 mol L^–1^; pH 4.7 ± 0.5]. Recovery of Sr and Pb was considered adequate at ratios of *c*_DGT_ values to bulk solution concentration (*c*_soln_) between 0.85 and 1.15.^28,29^ Details of the solution preparation and analysis are provided in the Supporting Information.

### Investigation of Time-Dependent Accumulation and Isotopic Fractionation

The analyte accumulation and possible isotopic fractionation over time were investigated by deploying triplicate TK100 DGT devices for varying times (2–24 h) in 3.5 L of well-stirred NaNO_3_ solution (*c* = 0.01 mol L^–1^) spiked with Sr [*c* = 50 mg L^–1^; *δ*_SRM 987_(^87^Sr/^86^Sr) = −3.74 ± 0.30‰] at pH 6.6 ± 0.1. After removal and DGT disassembly, TK100 membranes were subjected to single-step elution in HCl (*c* = 6 mol L^–1^). Details of the solution preparation and analysis are provided in the Supporting Information.

### Performance in Synthetic Soil Solution

TK100 DGT devices were deployed in triplicates for 24 h in 3.5 L of synthetic soil solutions to evaluate the performance of the technique under competitive conditions. The elemental composition of the synthetic soil solutions was based on typical literature values and is presented in Table S2. To test competitive effects of Ca, three different levels of Ca competition were included, thus giving three different immersion solutions “A”, “B”, and “C”. The ionic strength was balanced at 0.013 mol L^–1^ using Na^+^ as the counterion. To further test whether TK100 DGT sampling enables accurate isotope ratio measurement and source tracing under competitive conditions, solutions “A”, “B”, and “C” were spiked with three different Sr isotope standard solutions. These solutions were prepared by mixing the Sr(NO_3_)_2_ stock solution [*δ*_SRM 987_(^87^Sr/^86^Sr) = −3.71 ± 0.25‰] with SRM 987 [*δ*_SRM 987_(^87^Sr/^86^Sr) = 0.00 ± 0.37‰] to give three significantly different *δ*_SRM 987_(^87^Sr/^86^Sr) values in solution [“A”: *δ*_SRM 987_(^87^Sr/^86^Sr) = −3.71 ± 0.25‰ “B”: *δ*_SRM 987_(^87^Sr/^86^Sr) = −2.34 ± 0.25‰; “C”: *δ*_SRM 987_(^87^Sr/^86^Sr) = −1.39 ± 0.26‰]. All solutions were prepared over two days under constant stirring to ensure equilibration before DGT deployment.

### Application in Soils

Three contrasting mineral soils (0–30 cm depth), one from an agricultural site and two from metal-contaminated sites, were used. The agricultural soil was an acidic cambisol with a sandy loam texture from Siebenlinden, AT (“SL”), and has been previously characterized by Oburger et al.^30^ The two contaminated soils included an acidic cambisol with a loamy texture from Arnoldstein, AT (“AC”), and a calcic cambisol with a loamy texture from Mežica, SI (“MC”). These soils originate from former Pb mining areas and have been previously characterized by Lestan et al.^31^ The soils were air-dried, sieved to ≤2 mm, and stored at room temperature in plastic zip-lock bags until use. Details on DGT deployment, soil solution sampling, and NH_4_NO_3_ (*c* = 1 mol L^–1^) extraction and sample preparation are provided in the Supporting Information.

## Results and Discussion

### Analyte Uptake and Elution Recovery

The average uptake efficiencies, *f*_u_, over all mass balance experiments were 1.00 ± <0.01 for Sr and 0.98 ± 0.01 for Pb. This demonstrated quantitative sampling of Sr and Pb by TK100 membranes. The average elution recoveries, *f*_e_, were 0.95 ± 0.02 for Sr and 0.82 ± 0.04 for Pb after single-step elution of TK100 membranes for 24 h in HCl (*c* = 6 mol L^–1^) (Table S3; elution scheme 1). These values are consistent with previously reported Sr and Pb recoveries for TK100 column-type separations^20,25^ and indicate adequate elution recovery of Sr and Pb for isotope ratio measurements by MC ICP-MS.^21^ The low combined uncertainties (*u*_c_) on these values demonstrate the high intermediate precision and reproducibility of the analyte uptake and recovery (calculated as *SD* of within and between batch variation of TK100 membranes).

### Analyte Selectivity and Chromatographic Separation

TK100 has been shown to be selective for Sr in the presence of major matrix cations including Na^+^, Mg^2+^, K^+^, and Ca^2+^.^20^ However, to date, no information is available on the adsorption of Rb and Hg, which represent critical isobaric interferences in Sr and Pb isotope ratio measurements by MC ICP-MS.^21^ To determine if TK100 membranes also adsorb these elements, they were deployed in solutions containing Ca, Rb, and Hg along with Sr and Pb to ensure competitive conditions. Despite quantitative uptake of Sr (*f*_u_ = 0.99 ± 0.01) and Pb (*f*_u_ = 0.99 ± 0.01) (mean ± *u*_c_), significant co-uptake of Ca (*f*_u_ = 0.91 ± <0.01), Rb (*f*_u_ = 0.45 ± 0.01), and Hg (*f*_u_ = 0.98 ± <0.01) was observed (mean ± 1*SD*, *n* = 4). Therefore, the ability of the TK100 membranes for chromatographic separation of Ca, Rb, and Hg from Sr and Pb was further investigated by applying different eluents. Application of HNO_3_ (*c* = 8 mol L^–1^) as a matrix wash in the first elution step as recommended for column-type TK100 separations by Surman et al.^20^ resulted in high losses of Sr and Pb (Table S3; elution scheme 2) and consequently inadequate recoveries for isotope ratio measurements.^21^ Using HCl (*c* = 0.01 mol L^–1^) in the first elution step, cobound Ca and Rb were effectively removed, while the losses of both Sr and Pb were significantly reduced as compared to the original elution scheme (Table S3; elution scheme 3). However, Hg remained strongly retained (*f*_e_ = 0.02 ± <0.01), indicating its high affinity for the TK100 membrane as for the conventional Chelex gel.^32^ Hence, direct matrix-free sampling of Pb by TK100 DGT for isotope ratio analysis involving ^204^Pb is limited and eventually requires correction for residual ^204^Hg. In the second elution step, Sr (*f*_e_ = 0.75 ± 0.03) and Pb (*f*_e_ = 0.31 ± 0.01) were eluted in HCl (*c* = 2 mol L^–1^). Finally, residual Sr (*f*_e_ = 0.01 ± <0.01) and the main part of Pb (*f*_e_ = 0.56 ± 0.02) were eluted in the third elution step using HCl (*c* = 6 mol L^–1^). This elution scheme (3) was applied in all further experiments where Sr and Pb were sampled by DGT under competitive conditions. As Pb recoveries in the individual eluate fractions were low, samples of the HCl eluates from elution steps two and three were combined (φ = 1) for MC ICP-MS analysis, giving an overall Pb recovery of 0.87 ± 0.02.

### Blanks, Detection Limits, and Quantification Limits

The average blank values were 1.32 ± 1.23 ng disc^–1^ for Sr (*n* = 13) and 15.9 ± 2.9 ng disc^–1^ for Pb (*n* = 10). This equates to average *MDL*s and *MQL*s of 0.21 and 0.69 μg L^–1^ for Sr, respectively, and 0.38 and 1.28 μg L^–1^ for Pb, respectively, for a DGT deployment of 24 h at 25 °C. The *MDL* for Sr was thus three times lower than previously reported for Chelex DGT^18^ and also substantially lower than the minimum concentrations of Sr in soil solutions of highly weathered tropical soils (1 μg L^–1^).^33^ However, the *MDL* for Pb was 19 times higher than for Chelex DGT,^18^ indicating Pb contamination of the TK100 membrane. While this is not relevant for the most important application of the technique, *i.e.*, Pb-contaminated soils where Pb concentrations in soil solutions of >100 μg L^–1^ are found,^11^ it may limit Pb measurements in natural noncontaminated soils where Pb concentrations in soil solutions are often <1 μg L^–1^.^34^ Therefore, precleaning of TK100 membranes in HCl (*c* = 6 mol L^–1^) for 24 h was performed, which lowered the *MDL* for Pb by 1.5 times down to 0.25 μg L^–1^. Yet, the *MDL* was still 12.5 times higher than for Chelex DGT. This indicated that the main blank issue was related to the TK100 itself, as previously reported for the conventional crown-ether-based Sr spec resin.^35^ Further precleaning of the TK100 prior to embedment in the PTFE membrane may be required to enable Pb measurements at ultratrace levels.

### Diffusion Coefficients

**Figure 1:**
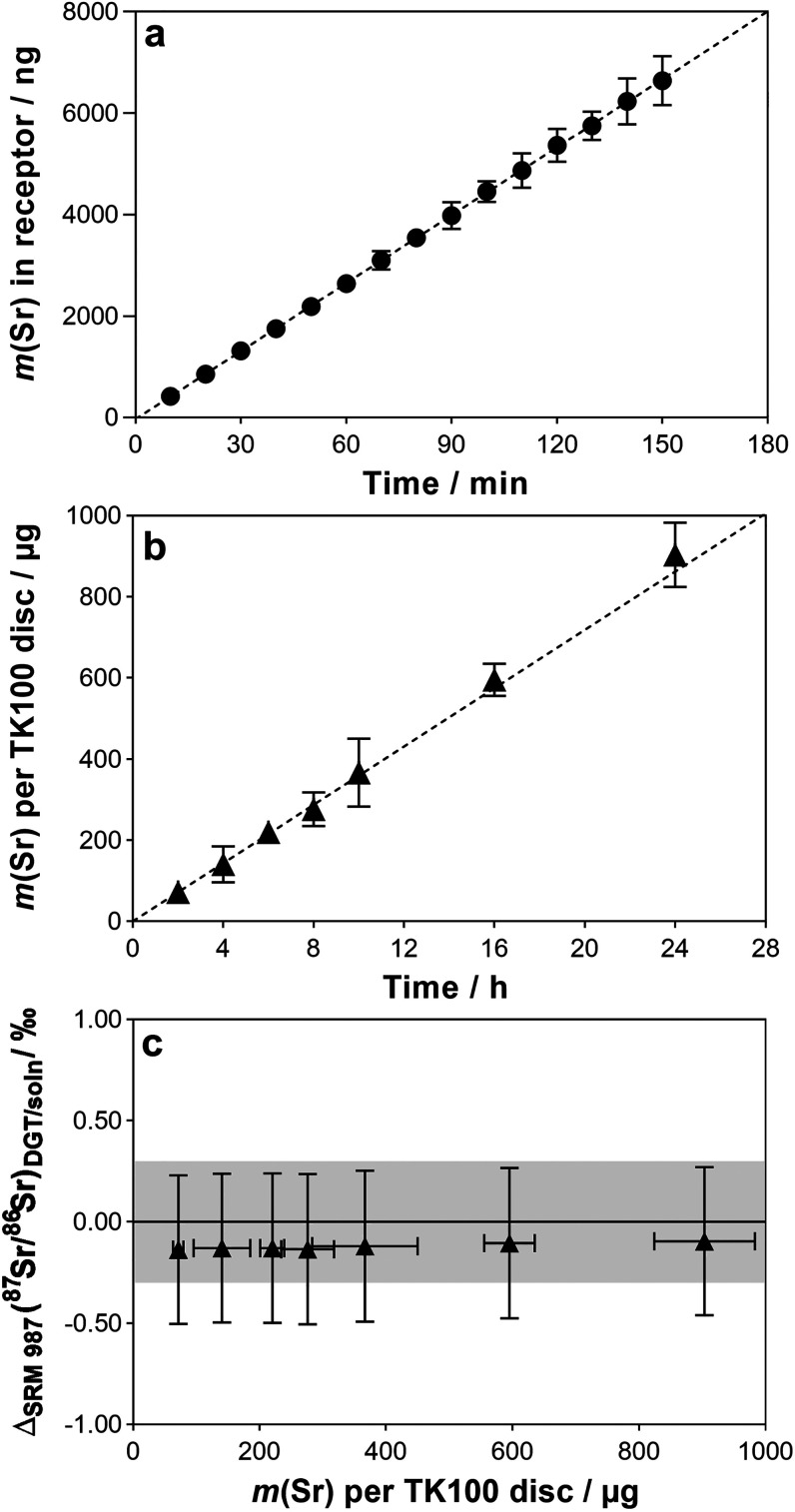
(a) Mass of Sr in receptor solution of the diffusion cell against time at 22 °C. The dashed line shows the linear regression (*R*^2^ > 0.99). (b) Mass of Sr per TK100 disc in DGT devices with increasing deployment time in solution. The dashed line shows the theoretical uptake line calculated using eq 2. (c) *Δ*_SRM 987_(^87^Sr/^86^Sr)_DGT/soln_ with increasing Sr mass on TK100 discs. Error bars and gray areas in the plots show expanded uncertainties (*U*, *k* = 2).

The mass of Sr that diffused with time from the source into the receptor solution through the APA gel is shown in Figure 1a. The resulting diffusion coefficient of Sr at 25 °C (*D*_25_) calculated from the regression line slope (0.74 ng s^–1^) was 6.17 × 10^–6^ ± 0.58 × 10^–6^ cm^2^ s^–1^. This value is 23% lower compared to the *D*_25_ of Sr in water (7.94 × 10^–6^ cm^2^ s^–1^),^36^ confirming previous works showing restricted diffusion of cationic solutes in APA gels.^37,38^ However, Chang et al.^17^ reported a *D*_25_ for Sr in an APA gel of 7.72 × 10^–6^ cm^2^ s^–1^, *i.e.*, indicating almost free diffusion of Sr in the gel. The apparent difference is likely caused by a different composition of the APA gel used by Chang et al.^17^ as compared to those that are now available commercially and routinely used. Changes in the manufacturing process of the agarose derivative cross-linker have resulted in gels with slightly smaller pore sizes,^37^ causing a more tortuous diffusion pathway through the polyacrylamide matrix and thus slower diffusivity. Diffusion coefficients of Sr and Pb in APA gels from 1 to 35 °C are provided in Table S4. These values were used for all calculations in this study.

### Effect of pH

Concentrations of Sr measured by TK100 DGT (*c*_DGT_) were in excellent agreement with those in solutions (*c*_soln_) in the pH range of 3.9–8.2 with an average *c*_DGT_/*c*_soln_ ratio of 1.00 ± 0.03 (mean ± 1*SD*; Table 1). This shows that Sr concentration measurements by TK100 DGT are independent of typical pH values found in natural soils. For Pb, *c*_DGT_/*c*_soln_ was well between 0.85 and 1.15 for all pH conditions, except for pH 7.5 (Table 1). In this solution, *c*_soln_ of Pb was only 34% at the start of DGT deployment compared to the average *c*_soln_ in all other solutions and decreased further by 55% over the 24 h deployment period. This observation indicates specific interaction between Pb and the 3-(morpholin-4-yl)propane-1-sulfonic acid (MOPS) buffer exclusively used in the solution with pH 7.5, leading to a substantial and continuing decrease of kinetically labile Pb in solution. It is possible that, at pH 7.5, deprotonation of the heterocyclic nitrogen atom in the morpholine ring of MOPS caused Pb complexation, as recently shown by isothermal titration colorimetry.^39^ Yet, it is unlikely that the dissociation rate constant of these complexes is sufficiently low to significantly limit the DGT uptake of Pb. More likely, Pb complexation by MOPS enhanced Pb adsorption to the plastic container walls at pH 7.5, which has been shown to also occur even at slightly acidic pH of 6.0.^40^ Although further research may be required to fully explain this observation, it is important to note that a direct pH effect is implausible, as *c*_DGT_/*c*_soln_ of Sr was close to 1 also at pH 7.5, while both Sr and Pb were sampled quantitatively under all other pH conditions.

**Table 1:** Effects of pH and Ionic Strength on Sr and Pb Sampling by TK100 DGT^a^

	Sr	Pb
pH	*c*_DGT_/*c*_soln_	
3.9	1.03 ± 0.12	1.04 ± 0.13
4.8	1.04 ± 0.13	1.06 ± 0.14
6.5	1.00 ± 0.12	1.06 ± 0.13
7.5	0.98 ± 0.13	0.68 ± 0.11
8.2	0.96 ± 0.13	0.95 ± 0.14
ionic strength (mol L^–1^)		
0.001	1.15 ± 0.35	1.18 ± 0.21
0.01	1.02 ± 0.13	1.01 ± 0.13
0.1	0.90 ± 0.11	0.94 ± 0.13
0.75	0.51 ± 0.15	0.71 ± 0.19

a Errors are expanded uncertainties (*U*, *k* = 2).

### Effect of Ionic Strength

The *c*_DGT_/*c*_soln_ ratios of both Sr and Pb were generally within 0.85–1.15 in solutions with ionic strengths of 0.001–0.1 mol L^–1^ (Table 1), which is the range found in natural porewaters of agricultural, metal-contaminated, and slightly to moderately saline soils.^34,41,42^ Thus, the TK100 DGT is applicable for use in natural soil environments with respect to the ionic strength. The performance of the TK100 DGT was, however, affected at very low and very high ionic strengths. At 0.001 mol L^–1^, the uncertainty on both Sr and Pb increased significantly and the measured *c*_DGT_/*c*_soln_ of Pb was slightly higher than 1.15. Conversely, at 0.75 mol L^–1^, which was included to test the method’s potential for application in seawater environments,^43^ the measured *c*_DGT_/*c*_soln_ of both Sr and Pb was evidently lower than 0.85. This is consistent with previous DGT works using different binding layers^28,44,45^ and can be explained by changes in the effective diffusion coefficient because of charge (at *I* ≤ 0.001 mol L^–1^) and viscosity effects (at *I* > 0.1 mol L^–1^) in diffusive gels and solutions, respectively.

### Investigation of Time-Dependent Accumulation and Isotopic Fractionation

Time-dependent analyte accumulation and possible isotopic fractionation during incremental uptake were investigated by deploying TK100 DGTs for up to 24 h in solutions with high levels of Sr (*c* = 51.7 ± 0.4 mg L^–1^). Sr was chosen as a test element for the experiment given its generally higher abundance in natural soil solutions (Table S2), lower binding affinity of TK100,^20^ higher relative isotopic mass difference, and a smaller isotopic natural-abundance variation as compared to Pb, which implies that the determined accumulation and isotopic fractionation are conservative estimates that can be expected to be applicable to Pb as well.

Figure 1b shows that the TK100 DGT accumulated Sr linearly over time (*R*^2^ > 0.99). The DGT-measured masses, corrected for *f*_e_, were in excellent agreement with those predicted from eq 1 with an average *m*_measured_/*m*_predicted_ ratio of 0.99 ± 0.06 (mean ± 1*SD*). The maximum Sr mass loading was 904 μg (288 μg cm^–2^ at *A*_p_ = 3.14 cm^2^), which was still very close to the predicted value of 862 μg and, notably, 1.6 times higher than the total Sr capacity reported for a nonselective binding layer containing the general cation-exchange resin AG50W-X8.^17^ The isotopic fractionation of ^87^Sr/^86^Sr, expressed as *Δ*_SRM 987_(^87^Sr/^86^Sr)_DGT/soln_ (eq 4), with increasing Sr mass loadings on TK100 membranes is shown in Figure 1c. It is evident that no significant isotopic fractionation of ^87^Sr/^86^Sr occurred at Sr mass loadings between 72 μg and 904 μg, as all *Δ*_SRM 987_(^87^Sr/^86^Sr)_DGT/soln_ values were not significantly different from zero with respect to the uncertainty. The average expanded uncertainty (*U*, *k* = 2) of *δ*_SRM 987_(^87^Sr/^86^Sr)_DGT_ values was 0.26‰, with MC ICP-MS analysis being the main contributor (85%). These results confirmed that (i) there is sufficiently rapid uptake and high total binding capacity by the TK100 membrane to ensure zero-sink sampling of Sr, and (ii) that the TK100 DGT is an appropriate passive sampler for the accurate and precise assessment of ^87^Sr/^86^Sr isotope ratios over the investigated time scale and conditions used in this experiment.

### Performance in Synthetic Soil Solution

The performance of the TK100 DGT was tested in synthetic soil solutions spiked with Sr (*c* = 275 μg L^–1^) and Pb (*c* = 15 μg L^–1^) as well as different levels of Ca to encompass environmentally relevant competition conditions (Table S2). As for the time-series experiment, Sr was chosen for evaluating the isotopic fractionation, given the higher relative isotopic mass difference and smaller natural-abundance variation as compared to Pb and hence the increased susceptibility to mass-dependent fractionation effects. The Sr concentrations and ^87^Sr/^86^Sr isotope ratios assessed by TK100 DGT and in the immersion solutions “A”, “B”, and “C” are shown in Table 2. DGT-measured Pb concentrations are provided in the Supporting Information (Table S5).

**Table 2:** DGT-Measured Solution Concentrations (*c*_DGT_), Bulk Solution Concentrations (*c*_soln_), *c*_DGT_/*c*_soln_ Ratios, DGT-Measured Isotope Ratios [*δ*_SRM 987_(^87^Sr/^86^Sr)_DGT_], Isotope Ratios in Bulk Solution [*δ*_SRM 987_ (^87^Sr/^86^Sr)_soln_], and Isotopic Difference between DGT- and Bulk Solution Isotope Ratios [*Δ*_SRM 987_(^87^Sr/^86^Sr)_DGT/soln_] of Sr in Synthetic Soil Solutions^a^

		Sr concentration			^87^Sr/^86^Sr isotope ratio		
solution	*c*(Ca) (mg L^–1^)	*c*_DGT_ (μg L^–1^)	*c*_soln_ (μg L^–1^)	*c*_DGT_/*c*_soln_	*δ*_SRM 987_ (^87^Sr/^86^Sr)_DGT_ (‰)	*δ*_SRM 987_ (^87^Sr/^86^Sr)_soln_ (‰)	*Δ*_SRM 987_ (^87^Sr/^86^Sr)_DGT/soln_ (‰)
A	51.4 ± 1.6	254 ± 48	259 ± 13	0.98 ± 0.19	–3.67 ± 0.28	–3.71 ± 0.25	0.03 ± 0.37
B	112 ± 10	272 ± 36	289 ± 24	0.94 ± 0.15	–2.41 ± 0.26	–2.39 ± 0.25	–0.01 ± 0.36
C	159 ± 6	252 ± 37	282 ± 18	0.86 ± 0.14	–1.46 ± 0.26	–1.39 ± 0.26	–0.07 ± 0.37

a Errors are expanded uncertainties (*U*, *k* = 2). The composition of the synthetic soil solution matrix is provided in Table S2.

The results showed that competition effects are likely to be negligible in typical soil solutions, with all *c*_DGT_/*c*_soln_ ratios of Sr and Pb between 0.85 and 1.15 (Tables 2 and S5). Only at a Ca level of 159 mg L^–1^, which confines the upper range typically encountered in arable soil solutions,^34^ the uptake of Sr was slightly impaired, as evidenced by *c*_DGT_/*c*_soln_ ratios approaching 0.85. It is noteworthy that the performance for sampling Sr at the two upper levels of Ca was substantially improved by precleaning the TK100 membrane in HCl (*c* = 6 mol L^–1^) for 24 h followed by thorough rinsing and storage in water. Thereby, increased *c*_DGT_/*c*_soln_ ratios of Sr from 0.81 ± 0.14 to 0.94 ± 0.15 at 112 mg L^–1^ Ca and 0.72 ± 0.16 to 0.86 ± 0.14 at 159 mg L^–1^ Ca were obtained. The HDEHP in TK100 is a cation exchanger having some affinity for Ca at near-neutral pH, causing its uptake into the organic phase. Precleaning of the TK100 membrane in HCl increases protonation of the organic phase, thus effectively lowering the HDEHP affinity for Ca while maintaining high affinity for Sr and Pb even at pH 2 and 3.^20^ Therefore, the proposed precleaning procedure may be an effective strategy to improve the TK100 DGT binding efficiency for Sr when applied in soils with elevated Ca levels. Other elements with high affinity for TK100 (*i.e.*, Y, Ba, Bi, Ra, U, and Am)^20^ are unlikely to compete with Sr uptake, given their trace to ultratrace contents typically present in natural soils.^3^

In this experiment, three-step elution (Table S3; elution scheme 3) of TK100 membranes was applied after DGT sampling to achieve separation of Sr and Pb from cobound matrix cations (Mg^2+^, K^+^, Ca^2+^, and Rb^+^). The obtained average cation separation efficiency, expressed as the relative difference between the elemental mass concentration ratio in the synthetic soil solutions and in the DGT eluates, was ≥96% for Sr and 100% for Pb (Table S6). Therefore, TK100 DGT sampling in combination with the selective three-step elution procedure in HCl enables direct *in situ* matrix separation of Sr and Pb and is thus beneficial for Sr and Pb isotope ratio measurements by MC ICP-MS. Comparison of the isotopic data for Sr sampled by TK100 DGT and in the standard solutions confirmed that neither diffusive-based sampling nor selective elution caused fractionation of Sr isotopes, as *Δ*_SRM 987_(^87^Sr/^86^Sr)_DGT/soln_ values remained close to zero with low expanded uncertainty for all deployments (Table 2). This proves that the isotopic composition of labile Sr can be accurately and precisely assessed using the proposed method, even under highly competitive conditions.

### Application in Soils

The TK100 DGT was applied to three soils with different physicochemical characteristics for 24 h and compared to conventional approaches for bioavailable Sr and Pb concentrations and isotope ratio assessment in soil, *i.e.*, soil solution and NH_4_NO_3_ extraction, respectively. Results are presented in Table 3.

The *c*_DGT_ values of Sr ranged between 46.6 μg L^–1^ in acidic AC and 168 μg L^–1^ in calcic MC and were significantly and systematically lower than the total dissolved concentrations, *c*_soln_, in soil solution (Table S7), as shown by *c*_DGT_/*c*_soln_ ratios between 0.19 and 0.32. This indicates that the concentration of Sr in soil solution of the investigated soils is limited by diffusional transport and thus only partially maintained by resupply via desorption from soil solid phases.^13,46^ For Pb, the results of the concentration measurements by DGT were distinctly different. As expected, *c*_DGT_ values of Pb in the agricultural SL soil were significantly lower than in the metal-contaminated MC and AC soils (Table 3). The *c*_DGT_/*c*_soln_ ratios for Pb were consistently higher than those for Sr, confirming a high degree of kinetic lability and quick resupply from soil solid phases as previously determined in stable isotope dilution experiments,^11^ thus approaching steady-state conditions.^13^ For subsequent isotope ratio analysis by MC ICP-MS, Sr eluates were further subjected to column-type matrix separation using the Sr spec resin (TrisKem)^47^ due to a more complex matrix of real soil DGT eluates (screening showed elevated levels of barium and rare-earth elements). Pb eluates were analyzed without additional separation following dilution to 10 μg L^–1^ of Pb. To cope with this, we recommend that DGT eluates should always be screened to check if matrix loads are low enough to allow for direct isotope ratio analysis by MC ICP-MS.

The resulting isotope ratios of Sr measured in DGT eluates, *δ*_SRM 987_(^87^Sr/^86^Sr)_DGT_, were significantly different between all soils, ranging from 12.6‰ in SL to −1.43‰ in MC (Table 3), which reflected the different geological backgrounds where the soils were sampled from. For example, the high *δ*_SRM 987_(^87^Sr/^86^Sr)_DGT_ in SL results from weathering of the ancient granitic bedrock,^48^ releasing radiogenic ^87^Sr during soil formation. For Pb, only the metal-contaminated AC and MC soils were analyzed due to a predominant contribution (>60%) of the procedural blank to the total Pb sampled by DGT in the arable SL soil. The obtained *δ*_SRM 981_(^207^Pb/^206^Pb)_DGT_ values of AC and MC were not significantly different (Table 3), indicating a similar source of Pb originating from common carbonate-hosted Pb–Zn deposits typical for these areas.^49^ Overall, the DGT-measured Sr and Pb isotope ratios were in excellent agreement with the corresponding *δ* values in NH_4_NO_3_-extractable soil fractions (Tables 3 and S8), suggesting that both methods access similar pools of Sr and Pb in soils. This is of high relevance for the application of the TK100 DGT technique as a tool for tracing the (geographic) origin of agricultural products or sources of Sr and Pb contamination in the environment, where soil extraction using NH_4_NO_3_ has been indispensable in the past.^5,6,10^

**Table 3:** DGT-Measured Soil Solution Concentrations (*c*_DGT_), Ratios of *c*_DGT_ Values to Bulk Soil Solution Concentrations (*c*_soln_), DGT-Measured Isotope Ratios [*δ*_std_(*^i^*E/*^j^*E)_DGT_], and Isotopic Difference between DGT- and NH_4_NO_3_-Extractable Fractions [*Δ*_std_(*^i^*E/*^j^*E)_DGT/NH_4_NO_3__] of Sr and Pb in Natural Soil Samples^a^

	Sr				Pb			
soil sample	*c*_DGT_ (μg L^–1^)	*c*_DGT_/*c*_soln_	*δ*_SRM 987_ (^87^Sr/^86^Sr)_DGT_ (‰)	*Δ*_SRM 987_ (^87^Sr/^86^Sr)_DGT/NH_4_NO_3__ (‰)	*c*_DGT_ (μg L^–1^)	*c*_DGT_/*c*_soln_	*δ*_SRM 981_ (^207^Pb/^206^Pb)_DGT_ (‰)	*Δ*_SRM 981_ (^207^Pb/^206^Pb)_DGT/NH_4_NO_3__ (‰)
SL	57.8 ± 9.7	0.32 ± 0.08	12.6 ± 0.42	–0.29 ± 0.52	0.95	1.00	n.m.	n.m.
AC	46.6 ± 9.1	0.21 ± 0.07	0.17 ± 0.35	–0.16 ± 0.45	231 ± 36	0.64 ± 0.11	–67.0 ± 0.3	0.20 ± 0.49
MC	168 ± 25	0.19 ± 0.03	–1.43 ± 0.29	–0.18 ± 0.41	74.8 ± 15.9	0.69 ± 0.15	–67.3 ± 0.4	0.17 ± 0.44

a Errors are expanded uncertainties (*U*, *k* = 2). No errors are shown for Pb values in SL, as these were <*MQL*. n.m. = not measured.

## Conclusions

This work has evaluated a new method for the accurate assessment of the elemental and isotopic compositions of labile, bioavailable Sr and Pb in soils with low uncertainties. Using the selectivity of TK100 membranes together with the mechanistic sampling principle of DGT, concomitant information on elemental resupply dynamics and natural isotopic variations of Sr and Pb in soil was obtained for the first time. This method is a significant advancement in environmental analysis of Sr and Pb by allowing for simultaneous and time-integrated measurements of labile Sr and Pb fractions with adequate elution recovery and quantitative matrix reduction already in the sampling step, thereby possibly facilitating sample preparation for MC ICP-MS. The total capacity of the TK100 DGT for binding Sr is very high compared to all existing Sr DGT techniques, and it performs effectively in the range of pH, ionic strength, and cation competition of environmental interest. Our results also showed that neither diffusive-based sampling nor selective elution or mass loading effects caused isotopic fractionation. Although detection limits for Pb were high compared to previous DGT techniques, there is scope for substantial improvement by further precleaning of the TK100 itself, offering the potential for simultaneous analysis of Sr and Pb isotopes at ultratrace levels in the future. Application of the TK100 DGT to natural soils demonstrated its unique capability as a passive sampling tool for Sr and Pb analyses and source tracing for further understanding of biogeochemical cycles of Sr and Pb in soils.

## Supporting Information Available

Laboratory procedures and materials; instrumental setups and parameters for isotope ratio measurements; uptake efficiency of TK100 resin and gels for Sr; uncertainty estimation; diffusion cell experiment; solution preparation and analysis in pH and ionic strength experiments as well as time-dependent accumulation and isotopic fractionation experiments; composition of synthetic soil solutions; DGT deployment; soil solution sampling and NH_4_NO_3_ extraction; applied elution schemes and elution recoveries; diffusion coefficients; competition effects on Pb concentration measurements; matrix separation efficiency; elemental mass concentrations insoil solutions; and isotope ratios of Sr and Pb in NH_4_NO_3_ soil extracts (PDF)

## References

[ref1] Capo R. C.; Stewart B. W.; Chadwick O. A. Strontium isotopes as tracers of ecosystem processes: theory and methods. Geoderma 1998, 82, 197–225. 10.1016/S0016-7061(97)00102-X.

[ref2] Komárek M.; Ettler V.; Chrastný V.; Mihaljevič M. Lead isotopes in environmental sciences: A review. Environ. Int. 2008, 34, 562–577. 10.1016/j.envint.2007.10.005. 18055013

[ref3] Kabata-Pendias A. Trace Elements in Soils and Plants, 4th ed.; Taylor & Francis Group: USA, 2010.

[ref4] Li H.-B.; Chen K.; Juhasz A. L.; Huang L.; Ma L. Q. Childhood Lead Exposure in an Industrial Town in China: Coupling Stable Isotope Ratios with Bioaccessible Lead. Environ. Sci. Technol. 2015, 49, 5080–5087. 10.1021/es5060622. 25803404

[ref5] Zannella C.; Carucci F.; Aversano R.; Prohaska T.; Vingiani S.; Carputo D.; Adamo P. Genetic and geochemical signatures to prevent frauds and counterfeit of high-quality asparagus and pistachio. Food Chem. 2017, 237, 545–552. 10.1016/j.foodchem.2017.05.158. 28764033

[ref6] Bataille C. P.; Crowley B. E.; Wooller M. J.; Bowen G. J. Advances in global bioavailable strontium isoscapes. Palaeogeogr., Palaeoclimatol., Palaeoecol. 2020, 555, 109849 10.1016/j.palaeo.2020.109849.

[ref7] Oeser R. A.; von Blanckenburg F. Strontium isotopes trace biological activity in the Critical Zone along a climate and vegetation gradient. Chem. Geol. 2020, 558, 119861 10.1016/j.chemgeo.2020.119861.

[ref8] Wang Z.; Wade A. M.; Richter D. D.; Stapleton H. M.; Kaste J. M.; Vengosh A. Legacy of anthropogenic lead in urban soils: Co-occurrence with metal(loids) and fallout radionuclides, isotopic fingerprinting, and in vitro bioaccessibility. Sci. Total Environ. 2022, 806, 151276 10.1016/j.scitotenv.2021.151276. 34717995

[ref9] Wooller M. J.; Bataille C.; Druckenmiller P.; Erickson G. M.; Groves P.; Haubenstock N.; Howe T.; Irrgeher J.; Mann D.; Moon K.; Potter B. A.; Prohaska T.; Rasic J.; Reuther J.; Shapiro B.; Spaleta K. J.; Willis A. D. Lifetime mobility of an Arctic woolly mammoth. Science 2021, 373, 806–808. 10.1126/science.abg1134. 34385399

[ref10] Prohaska T.; Wenzel W. W.; Stingeder G. ICP-MS-based tracing of metal sources and mobility in a soil depth profile via the isotopic variation of Sr and Pb. Int. J. Mass Spectrom. 2005, 242, 243–250. 10.1016/j.ijms.2004.11.028.

[ref11] Degryse F.; Waegeneers N.; Smolders E. Labile lead in polluted soils measured by stable isotope dilution. Eur. J. Soil Sci. 2007, 58, 1–7. 10.1111/j.1365-2389.2005.00788.x.

[ref12] DIN ISO 19730 Bodenbeschaffenheit - Extraktion von Spurenelementen aus Böden mit Ammoniumnitratlösung (ISO 19730:2008). https://shop.austrian-standards.at/action/de/public/details/338432/DIN_ISO_19730_2009_07 (accessed November 08, 2021).

[ref13] Hooda P. S.; Zhang H.; Davison W.; Edwards A. C. Measuring bioavailable trace metals by diffusive gradients in thin films (DGT): soil moisture effects on its performance in soils. Eur. J. Soil Sci. 1999, 50, 285–294. 10.1046/j.1365-2389.1999.00226.x.

[ref14] Zhang H.; Davison W. Use of diffusive gradients in thin-films for studies of chemical speciation and bioavailability. Environ. Chem. 2015, 12, 85–101. 10.1071/EN14105.

[ref15] Degryse F.; Smolders E. DGT and Bioavailability. In Diffusive Gradients In Thin-Films For Environmental Measurements;Davison W.; Zhang H., Eds.; Cambridge University Press: Cambridge, 2016; pp 216–262.

[ref16] Hanousek O.; Santner J.; Mason S.; Berger T. W.; Wenzel W. W.; Prohaska T. Diffusive gradients in thin films measurement of sulfur stable isotope variations in labile soil sulfate. Anal. Bioanal. Chem. 2016, 408, 8333–8341. 10.1007/s00216-016-9949-2. 27687185 PMC5116312

[ref17] Chang L.-Y.; Davison W.; Zhang H.; Kelly M. Performance characteristics for the measurement of Cs and Sr by diffusive gradients in thin films (DGT). Anal. Chim. Acta 1998, 368, 243–253. 10.1016/S0003-2670(98)00215-3.

[ref18] Garmo Ø. A.; Røyset O.; Steinnes E.; Flaten T. P. Performance Study of Diffusive Gradients in Thin Films for 55 Elements. Anal. Chem. 2003, 75, 3573–3580. 10.1021/ac026374n. 14570212

[ref19] Desaulty A.-M.; Méheut M.; Guerrot C.; Berho C.; Millot R. Coupling DGT passive samplers and multi-collector ICP-MS: A new tool to measure Pb and Zn isotopes composition in dilute aqueous solutions. Chem. Geol. 2017, 450, 122–134. 10.1016/j.chemgeo.2016.12.023.

[ref20] Surman J. J.; Pates J. M.; Zhang H.; Happel S. Development and characterisation of a new Sr selective resin for the rapid determination of 90Sr in environmental water samples. Talanta 2014, 129, 623–628. 10.1016/j.talanta.2014.06.041. 25127642

[ref21] Retzmann A.; Zimmermann T.; Pröfrock D.; Prohaska T.; Irrgeher J. A fully automated simultaneous single-stage separation of Sr, Pb, and Nd using DGA Resin for the isotopic analysis of marine sediments. Anal. Bioanal. Chem. 2017, 409, 5463–5480. 10.1007/s00216-017-0468-6. 28674822

[ref22] Horsky M.; Irrgeher J.; Prohaska T. Evaluation strategies and uncertainty calculation of isotope amount ratios measured by MC ICP-MS on the example of Sr. Anal. Bioanal. Chem. 2016, 408, 351–367. 10.1007/s00216-015-9003-9. 26472320 PMC4709391

[ref23] Zhang H.; Davison W. Performance Characteristics of Diffusion Gradients in Thin Films for the in Situ Measurement of Trace Metals in Aqueous Solution. Anal. Chem. 1995, 67, 3391–3400. 10.1021/ac00115a005.

[ref24] Linsinger T. Application Note 1 - Comparison of a measurement result with the certified value. https://ec.europa.eu/jrc/sites/default/files/erm_application_note_1_en.pdf(accessed October 08, 2021).

[ref25] Dirks C.; Surman J. J.; Pates J. M.; Happel S. Rapid determination of Pb-210 and Sr-90 in water samples using new crown-ether based extraction chromatographic resins. https://www.triskem-international.com/scripts/files/59d0aa2e1fe2d6.11232404/8_rapid_determination_of_pb-210_and_sr-90_in_water_samples_using_new_crown-ether_based_extraction_chromatographic_resins.pdf (accessed December 20, 2021).

[ref26] van Es E.; Russell B. C.; Ivanov P.; Garcia Miranda M.; Read D.; Dirks C.; Happel S. The behaviour of 226Ra in high-volume environmental water samples on TK100 resin. J. Radioanal. Nucl. Chem. 2017, 312, 105–110. 10.1007/s10967-017-5203-4. 28366971 PMC5357471

[ref27] DGT Research Diffusion Coefficients. https://www.dgtresearch.com/diffusion-coefficients (accessed January 29, 2020).

[ref28] French M. A.; Zhang H.; Pates J. M.; Bryan S. E.; Wilson R. C. Development and Performance of the Diffusive Gradients in Thin-Films Technique for the Measurement of Technetium-99 in Seawater. Anal. Chem. 2005, 77, 135–139. 10.1021/ac048774b. 15623288

[ref29] Bennett W. W.; Teasdale P. R.; Panther J. G.; Welsh D. T.; Jolley D. F. Speciation of dissolved inorganic arsenic by diffusive gradients in thin films: selective binding of AsIII by 3-mercaptopropyl-functionalized silica gel. Anal. Chem. 2011, 83, 8293–8299. 10.1021/ac202119t. 21967720

[ref30] Oburger E.; Gruber B.; Schindlegger Y.; Schenkeveld W. D. C.; Hann S.; Kraemer S. M.; Wenzel W. W.; Puschenreiter M. Root exudation of phytosiderophores from soil-grown wheat. New Phytol. 2014, 203, 1161–1174. 10.1111/nph.12868. 24890330 PMC4143957

[ref31] Lestan D. Novel chelant-based washing method for soil contaminated with Pb and other metals: A pilot-scale study. Land Degrad. Dev. 2017, 28, 2585–2595. 10.1002/ldr.2818.

[ref32] Dočekalová H.; Diviš P. Application of diffusive gradient in thin films technique (DGT) to measurement of mercury in aquatic systems. Talanta 2005, 65, 1174–1178. 10.1016/j.talanta.2004.08.054. 18969928

[ref33] Pett-Ridge J. C.; Derry L. A.; Barrows J. K. Ca/Sr and 87Sr/86Sr ratios as tracers of Ca and Sr cycling in the Rio Icacos watershed, Luquillo Mountains, Puerto Rico. Chem. Geol. 2009, 267, 32–45. 10.1016/j.chemgeo.2008.11.022.

[ref34] Amelung W.; Blume H.-P.; Fleige H.; Horn R.; Kandeler E.; Kögel-Knabner I.; Kretzschmar R.; Stahr K.; Wilke B.-M. Scheffer/Schachtschabel Lehrbuch der Bodenkunde, 17th ed.; Springer Spektrum, 2018.

[ref35] Deniel C.; Pin C. Single-stage method for the simultaneous isolation of lead and strontium from silicate samples for isotopic measurements. Anal. Chim. Acta 2001, 426, 95–103. 10.1016/S0003-2670(00)01185-5.

[ref36] Yuan-Hui L.; Gregory S. Diffusion of ions in sea-water and in deep-sea sediments. Geochim. Cosmochim. Acta 1974, 38, 703–714. 10.1016/0016-7037(74)90145-8.

[ref37] Scally S.; Davison W.; Zhang H. Diffusion coefficients of metals and metal complexes in hydrogels used in diffusive gradients in thin films. Anal. Chim. Acta 2006, 558, 222–229. 10.1016/j.aca.2005.11.020.

[ref38] Shiva A. H.; Teasdale P. R.; Bennett W. W.; Welsh D. T. A systematic determination of diffusion coefficients of trace elements in open and restricted diffusive layers used by the diffusive gradients in a thin film technique. Anal. Chim. Acta 2015, 888, 146–154. 10.1016/j.aca.2015.07.027. 26320970

[ref39] Xiao C.-Q.; Huang Q.; Zhang Y.; Zhang H.-Q.; Lai L. Binding thermodynamics of divalent metal ions to several biological buffers. Thermochim. Acta 2020, 691, 178721 10.1016/j.tca.2020.178721.

[ref40] Warnken K. W.; Zhang H.; Davison W. Performance characteristics of suspended particulate reagent-iminodiacetate as a binding agent for diffusive gradients in thin films. Anal. Chim. Acta 2004, 508, 41–51. 10.1016/j.aca.2003.11.051.

[ref41] Hoefer C.; Santner J.; Borisov S. M.; Wenzel W. W.; Puschenreiter M. Integrating chemical imaging of cationic trace metal solutes and pH into a single hydrogel layer. Anal. Chim. Acta 2017, 950, 88–97. 10.1016/j.aca.2016.11.004. 27916135

[ref42] Shahid S. A.; Zaman M.; Heng L. Introduction to Soil Salinity, Sodicity and Diagnostics Techniques. In Guideline for Salinity Assessment, Mitigation and Adaptation Using Nuclear and Related Techniques; Springer International Publishing: Cham, 2018; Vol. 26, pp 1–42.

[ref43] Grasshoff K.; Ehrhardt M.; Kremling K.; Anderson L. Methods of Seawater Analysis, 3rd ed.; Wiley-VCH: New York, 1999.

[ref44] la Torre M. C. A.-D.; Beaulieu P.-Y.; Tessier A. In situ measurement of trace metals in lakewater using the dialysis and DGT techniques. Anal. Chim. Acta 2000, 418, 53–68. 10.1016/S0003-2670(00)00946-6.

[ref45] Sangi M. R.; Halstead M. J.; Hunter K. A. Use of the diffusion gradient thin film method to measure trace metals in fresh waters at low ionic strength. Anal. Chim. Acta 2002, 456, 241–251. 10.1016/S0003-2670(02)00012-0.

[ref46] Ernstberger H.; Zhang H.; Tye A.; Young S.; Davison W. Desorption Kinetics of Cd, Zn, and Ni Measured in Soils by DGT. Environ. Sci. Technol. 2005, 39, 1591–1597. 10.1021/es048534d. 15819214

[ref47] Irrgeher J.; Prohaska T.; Sturgeon R. E.; Mester Z.; Yang L. Determination of strontium isotope amount ratios in biological tissues using MC-ICPMS. Anal. Methods 2013, 5, 1687–1694. 10.1039/c3ay00028a.

[ref48] Geological Survey of Austria Geological Map 1:50,000. https://www.geologie.ac.at/en/research-development/mapping/geology/geological-map-150000 (accessed January 23, 2022).

[ref49] Melcher F.; Onuk P. Potential of Critical High-technology Metals in Eastern Alpine Base Metal Sulfide Ores. BHM Berg Hüttenmännische Monatsh. 2019, 164, 71–76. 10.1007/s00501-018-0818-5.

