# Supplementary material for: Selective Diffusive Gradients in Thin Films (DGT) for the Simultaneous Assessment of Labile Sr and Pb Concentrations and Isotope Ratios in Soils
Source: Anal Chem. 2022 Apr 15;94(16):6338–46. doi: 10.1021/acs.analchem.2c00546 (PMC9047413; doi:10.1021/acs.analchem.2c00546)
Supplement: Supplementary file 1 — ac2c00546_si_001.pdf [file ac2c00546_si_001.pdf]

Supporting Information

Selective Diffusive Gradients in Thin Films (DGT) for the Simultaneous Assessment of Labile Sr and Pb Concentrations and Isotope Ratios in Soils

Stefan Wagner[a,b], Jakob Santner[a,c], Johanna Irrgeher[a], Markus Puschenreiter[d], Steffen Happel[e], and Thomas Prohaska[a,*]

[a]Department General, Analytical and Physical Chemistry, Chair of General and Analytical Chemistry, Montanuniversität Leoben, Franz-Josef-Strasse 18, 8700, Leoben, Austria

[b]Department of Chemistry, Institute of Analytical Chemistry, University of Natural Resources and Life Sciences, Vienna, Konrad-Lorenz-Strasse 24, 3430, Tulln, Austria

[c]Department of Crop Sciences, Institute of Agronomy, University of Natural Resources and Life Sciences, Vienna, Konrad-Lorenz-Strasse 24, 3430, Tulln, Austria

[d]Department of Forest and Soil Sciences, Institute of Soil Research, University of Natural Resources and Life Sciences, Vienna, Konrad-Lorenz-Strasse 24, 3430, Tulln, Austria

[e]TrisKem International, 3 Rue des Champs Géons, ZAC de l'Eperon, 35170 Bruz, France

[*]Corresponding author: thomas.prohaska@unileoben.ac.at

Contents	Page

**Experimental Section**	S-3
Laboratory procedures and materials	S-3
Table S1. Instrumental setups and parameters for Sr and Pb isotope ratio measurements	S-4
Figure S1. Uptake efficiency of TK100 resin and gels for Sr	S-5
Uncertainty estimation	S-6
Diffusion cell experiment	S-7
Solution preparation in pH and ionic strength experiments	S-8
Solution preparation and analysis in time-dependent accumulation and isotopic fractionation experiments	S-9
Table S2. Composition of synthetic soil solutions	S-10
DGT deployment, soil solution sampling, and NH_4NO_3 extraction	S-11
**Results and Discussion**	S-12
Table S3. Applied elution schemes and elution recoveries	S-12
Table S4. Diffusion coefficients of Sr and Pb in APA diffusive gel	S-13
Table S5. DGT-measured solution concentrations (c_{DGT}), bulk solution concentrations (c_{soln}), and ratios of c_{DGT} to c_{soln} values of Pb in synthetic soil solutions	S-14
Table S6. Efficiency of TK100 DGT for separation of Sr and Pb from different potentially interfering matrix cations	S-15
Table S7. Elemental mass concentrations in soil solutions of the experimental soils	S-16
Table S8. Isotope ratios of Sr and Pb in NH_4NO_3 (c = 1 mol L^{-1}) extracts of the experimental soils	S-17

Experimental Section

To comply with notation conventions used in DGT literature, the symbol c is used throughout this work for both molar and mass concentrations in solution instead of the designated symbols c and γ recommended by the International Union of Pure and Applied Chemistry (IUPAC).

Laboratory procedures and materials

Polyethylene (PE) vials, containers, test tubes, and pipette tips, as well as piston-type acrylonitrile butadiene styrene (ABS) DGT moldings (R-SDU; DGT Research Ltd., UK) were pre-cleaned in a two-stage washing procedure with dilute HNO_3 (w = 10% and w = 1%, respectively; p.a., Merck, DE), rinsed with laboratory water type 1 (0.055 µS cm^{-1}; TKAGenPure, Thermo Electron LED GmbH, DE) and dried in a metal-free class 100 laminar flow cabinet. Laboratory water type 1 was used for preparing all experimental solutions. Laboratory water type 1, as well as concentrated HCl (w = 37 %; trace metals basis, PanReac AppliChem, DE) and HNO_3 (w = 65 %; p.a., Merck, DE) were further purified by sub-boiling distillation (DST-100, Savillex, USA) and used for pre-cleaning, conditioning, rinsing, storage, and elution of TK100 membranes as well as all ICP-MS analyses. Wherever possible, handling and preparation of DGT components and samples was performed in a laminar flow cabinet (Clean Air, EuroFlow EF/S, Telstar Laboratory Equipment B.V., NL). Chemical reagents of analytical grade or higher were used in all experiments. Single element stock solutions were prepared by dissolving $Sr(NO_3)_2$ (Merck), $Pb(NO_3)_2$ (Merck), $NaNO_3$ (Merck), $Mg(NO_3)_2$ (Merck), KNO_3 (VWR, AT), $Ca(NO_3)_2 \times 4\ H_2O$ (Merck), RbCl (Fluka, CH), $HgCl_2$ (Alfa Aesar, USA), and NH_4NO_3 (VWR). These stocks were used for preparing the experimental single- or multielement solutions. Certified isotopic reference standards were obtained from the National Institute of Standards and Technology, USA (NIST SRM 987, $SrCO_3$; NIST SRM 981, ≥99.9 % purity lead metal).

Table S1. Instrumental setups and parameters for Sr and Pb isotope ratio measurements

	Sr	Pb
Parameter	MC ICP-MS Nu Sapphire (SP017, Nu Instruments) [a]	
RF power	1300 W	
Coolant flow	13 L/min	
Auxiliary flow	0.9 L/min	
Nebulizer pressure	30 psi	
Interface cones	Nickel, dry	
Sample introduction system	Apex Omega with MicroFlow PFA-ST nebulizer (both ESI)	
Measurement mode	Static batch analysis (6 blocks with 10 measurement cycles with 10 s integration time)	
Resolution mode	$m/\Delta m$ = ~300	
Cup configuration	H10: ^{91}Zr; H8: ^{90}Zr; H6: ^{88}Sr; H4: ^{87}Sr; H2: ^{86}Sr; Ax: ^{85}Rb; L2: ^{84}Sr; L4: ^{83}Kr; L6: ^{82}Kr	H3: ^{208}Pb; H2: ^{207}Pb; H1: ^{206}Pb; Ax: ^{205}Tl; L1: ^{204}Pb; L2: ^{203}Tl; L3: ^{202}Hg
Axial mass / mass separation	85 / 0.5	205 / 1
Typical sensitivity	900 V/(µg g^{-1})	1300 V/(µg g^{-1})
Parameter	MC ICP-MS Nu Plasma HR (NP048, Nu Instruments) [b]	
RF power	1300 W	
Coolant flow	13 L/min	
Auxiliary flow	0.9 L/min	
Nebulizer pressure	26-31 psi	
Interface cones	Nickel, dry	
Sample introduction system	Aridus II (Teledyne CETAC) with MicroFlow PFA-ST nebulizer (ESI)	
Measurement mode	Static batch analysis (6 blocks with 10 measurement cycles with 10 s integration time)	
Resolution mode	$m/\Delta m$ = ~300	
Cup configuration	H6: ^{91}Zr; H5: ^{90}Zr; H2: ^{88}Sr; Ax: ^{87}Sr; L2: ^{86}Sr; L3: ^{85}Rb; L4: ^{84}Sr	H4: ^{208}Pb; H3: ^{207}Pb; H2: ^{206}Pb; H1: ^{205}Tl; Ax: ^{204}Pb; L1: ^{203}Tl; L2: ^{202}Hg
Axial mass / mass separation	87 / 0.5	204 / 1
Typical sensitivity	130 V/(µg g^{-1})	385 V/(µg g^{-1})

[a] Used for the following isotope ratio measurements: Sr in synthetic soil solutions; Sr in natural soil samples; Pb in synthetic soil solutions; Pb in natural soil samples

[b] Used for the following isotope ratio measurements: Investigation of time-dependent Sr isotopic fractionation; Sr in separated NH_4NO_3 soil extracts; Pb in separated NH_4NO_3 soil extracts

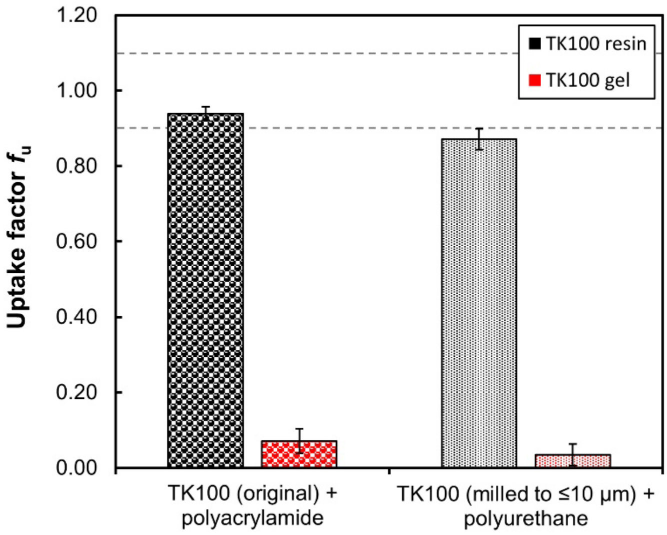

Figure S1. Uptake efficiency of TK100 resin and gels for Sr. Error bars show 1*SD* (n = 4). Experimental conditions: V = 10 mL, c(Sr) = 500 µg L^{-1}, pH = 5.5, c(NaCl) = 0.01 mol L^{-1}, t = 24 h.

Uncertainty estimation

Combined uncertainties (u_c) of the Sr and Pb uptake and elution factors were calculated as the square root of the sum of the squared standard deviations of the measurement repeatability ($n = 3$ for single-step elution; $n = 4$ for three-step elution) and reproducibility ($n = 2$). u_c of DGT-measured concentrations were calculated according to Kreuzeder et al.[1] and u_c of the DGT-measured isotope ratios were calculated according to Horsky et al.,[2] both including the DGT application repeatability ($n = 3$). Uncertainties provided on c_{DGT}/c_{soln} values were calculated based on the uncertainties of both c_{DGT} and c_{soln}. Uncertainties provided on $\Delta_{std}(^{i}E/^{j}E)_{DGT/ref}$ values were calculated based on the uncertainties of both $\delta_{std}(^{i}E/^{j}E)_{DGT}$ and $\delta_{std}(^{i}E/^{j}E)_{ref}$. The expanded uncertainty U was obtained by multiplying u_c with a coverage factor (k) of two.

Diffusion cell experiment

The diffusion cell was made of two 150 mL poly(methyl 2-methylpropenoate) compartments, each equipped with a circular 1.6 cm diameter opening. A 2.5 cm diameter APA diffusive gel with 0.8 mm thickness was placed between the openings and the compartments were clamped together. The compartments were filled with 100 mL of $NaNO_3$ solution (c = 0.01 mol L^{-1}) either with Sr (c = 5 mg L^{-1}; source solution) or without Sr (receptor solution). The source and receptor solutions were stirred continuously using magnetic stirrers. After 10 min of equilibration, 200 µL aliquots were taken from both reservoirs every 10 min for 2.5 h, acidified to give a dilute HCl (w = 2%) matrix and analyzed by ICP-MS. D(Sr) was calculated from the slope (ng s^{-1}) of the regression line and the mass concentration of Sr in the source solution as described previously.[3, 4] The diffusion cell experiment was repeated three times over the course of three different days to assess the within-lab reproducibility. The pH and temperature of both solutions was measured at the start and end of each experiment and remained constant (pH 5.3 ± 0.1; T = 22.3 °C ± 0.4 °C).

Solution preparation and analysis in pH and ionic strength experiments

To test the effect of pH on TK100 DGT performance for simultaneous sampling of Sr and Pb, TK100 DGT devices were deployed in solutions with pH 3.9, 4.8, 6.5, 7.5, and 8.2 at a constant $NaNO_3$ (c = 0.01 mol L^{-1}) background. The pH was adjusted using dilute HNO_3 or NaOH (both c = 1 mol L^{-1}). Solutions with pH 6.5, 7.5, and 8.2 were buffered with 2-morpholin-4-ylethanesulfonic acid (MES), 3-(Morpholin-4-yl)propane-1-sulfonic acid (MOPS), and 2-[4-(2-Hydroxyethyl)piperazin-1-yl]ethane-1-sulfonic acid (HEPES), respectively (all c = 0.05 mol L^{-1}), to ensure a stable pH during the deployment period. These solutions were further prepared with an ionic background of $NaNO_3$ (c = 0.001 mol L^{-1}) and $Mg(NO_3)_2$ (c = 0.004 mol L^{-1}) to reduce sorption of Pb to the container walls at elevated pH.[5] To test the effect of ionic strength, TK100 DGTs were deployed in solutions containing $NaNO_3$ at c = 0.001, 0.01, 0.1, and 0.75 mol L^{-1}. The pH of these (non-buffered) solutions decreased over the deployment time from pH ~5 to ~4 due to H^+ release from HDEHP following ion exchange with Sr^{2+} and Pb^{2+}. In both pH and ionic strength experiments, the Sr and Pb spikes were added 24 h before DGT deployment, the solution temperature (24.9 °C ± 0.9 °C) was monitored over the deployment period, and grab samples (n = 3) were analyzed by ICP-MS.

Solution preparation and analysis in time-dependent accumulation and isotopic fractionation experiments

To investigate the time-dependent analyte accumulation and possible isotopic fractionation, TK100 DGT devices were deployed for 2, 4, 6, 8, 10, 16, and 24 h in the deployment solution [c(Sr) = 50 mg L^{-1}; $\delta_{\mathrm{SRM\ 987}}(^{87}\mathrm{Sr}/^{86}\mathrm{Sr})$ = -3.74 ‰ ± 0.30 ‰; $c(\mathrm{NaNO_3})$ = 0.01 mol L^{-1}]. The pH of the deployment solution was adjusted and buffered to 6.6 ± 0.1 using NaOH (c = 1 mol L^{-1}) and MES (c = 0.05 mol L^{-1}), respectively. Grab samples were taken at each time point and analyzed for Sr concentrations by ICP-MS as well as for changes in Sr isotope ratios by MC ICP-MS. Eluates were measured by ICP-MS to determine the mass of Sr adsorbed on TK100 membranes. Moreover, $^{87}\mathrm{Sr}/^{86}\mathrm{Sr}$ ratios in DGT eluates, $\delta_{\mathrm{SRM\ 987}}(^{87}\mathrm{Sr}/^{86}\mathrm{Sr})_{\mathrm{DGT}}$, were measured by MC ICP-MS and compared to those in grab samples to evaluate possible isotopic fractionation effects. The solution temperature was monitored over the deployment period and remained stable at 23.1 °C ± 0.4 °C.

Table S2. Composition of synthetic soil solutions [a]

Parameter	Unit	Mean value(s) in synthetic soil solutions	Typical values in natural soil solutions	Reference
pH		6.0 [a]	3 - 8	6
I	mol L^{-1}	0.013	0.004 - 0.016 [b]	6
c(Mg)	mg L^{-1}	11.9	0.1 - 25	6
c(K)	mg L^{-1}	13.1	1 - 30	6
c(Ca)	mg L^{-1}	51; 112; 159	1 - 160	6
c(Rb)	µg L^{-1}	208	1 - 1000	7, 8
c(Sr)	µg L^{-1}	275	1 - 1000	9, 10, 11
c(Pb)	µg L^{-1}	15	<1 - 1000	6, 12

[a] pH was buffered using MES (c = 0.05 mol L^{-1}). [b] I was calculated from literature values of the electrical conductivity according to Alva et al.[13]

DGT deployment, soil solution sampling, and NH_4NO_3 extraction

For DGT deployment, ~150 g of each soil sample was mixed with laboratory water type 1 to reach 100 % of their maximum water holding capacity. The saturated soil pastes were incubated at 24 °C for 24 h and then applied as a ~2 mm-thick layer on the DGT devices (n = 3). The DGTs were then gently tapped a few times to ensure a complete contact between the filter surface of the sampler and the soil. DGT sampling was conducted in closed plastic boxes for 24 h at 24 °C in an incubator. After deployment, DGTs were washed to remove the adhering soil paste and disassembled. The TK100 discs were retrieved and subjected to three-step elution. Sub-samples (V = ~20 mL) of the incubated soil pastes were used to collect soil solution by centrifugation at 5000 × g for 15 min.[14] The supernatants were passed through a 0.45 µm-pore size PES syringe filter, immediately acidified to give a dilute HCl (w = 2 %) matrix, and then analyzed by ICP-MS. In parallel, NH_4NO_3 (c = 1 mol L^{-1}) extraction of soil samples was performed according to DIN ISO 19730.[15] The NH_4NO_3 extracts were diluted 20-fold and subjected to matrix separation using an automated, low-pressure chromatographic system (prepFAST-MC, ESI). Simultaneous separation of Sr and Pb was achieved using self-packed columns with 3 mL bed volume of DGA resin (TrisKem). Recoveries over all samples were ≥98 % for Sr and ≥71 % for Pb. A detailed description of the procedure can be found elsewhere.[16]

Results and Discussion

Table S3. Applied elution schemes and elution recoveries (expressed as elution factors f_e) determined in mass balance experiments [a]

Elution scheme	Analyte	HNO_3 (c = 8 mol L^{-1})	HCl (c = 0.01 mol L^{-1})	HCl (c = 2 mol L^{-1})	HCl (c = 6 mol L^{-1})	total
(1) single-step	Sr	-	-	-	0.95 ± 0.02	0.95 ± 0.02
	Pb	-	-	-	0.82 ± 0.04	0.82 ± 0.04
(2) three-step (not further used)	Sr	0.42 ± 0.01	-	0.56 ± 0.01	0.02 ± <0.01	1.00 ± 0.01
	Pb	0.27 ± 0.01	-	0.17 ± 0.02	0.47 ± 0.02	0.91 ± 0.01
(3) three-step	Sr	-	0.23 ± 0.03	0.75 ± 0.03	0.01 ± <0.01	0.99 ± 0.01
	Pb	-	0.04 ± 0.01	0.31 ± 0.01	0.56 ± 0.02	0.90 ± 0.01
	Ca	-	0.73 ± 0.01	0.30 ± 0.03	0.02 ± 0.01	1.05 ± 0.03
	Rb	-	0.90 ± 0.02	0.09 ± <0.01	*<LOD*	0.99 ± 0.02
	Hg	-	0.02 ± <0.01	0.58 ± 0.01	0.21 ± <0.01	0.81 ± 0.01

[a] Errors are combined uncertainties (u_c) for Sr and Pb in elution schemes (1) and (3), all others are 1SD (n = 4).

Table S4. Diffusion coefficients (D; × 10^{-6} cm^2 s^{-1}) of Sr and Pb in APA diffusive gel. D(Sr) was measured in a diffusion cell experiment and calculated for different temperatures between 1 °C and 35 °C. D(Pb) values were taken from DGT Research Ltd.[17]

Temperature (°C)	D(Sr)	D(Pb)
1	2.88	3.75
2	2.99	3.89
3	3.10	4.04
4	3.21	4.18
5	3.33	4.34
6	3.45	4.49
7	3.57	4.65
8	3.70	4.81
9	3.82	4.97
10	3.95	5.14
11	4.08	5.31
12	4.22	5.49
13	4.35	5.66
14	4.49	5.84
15	4.63	6.03
16	4.77	6.21
17	4.92	6.40
18	5.07	6.59
19	5.22	6.79
20	5.37	6.99
21	5.53	7.19
22	5.68	7.40
23	5.84	7.60
24	6.01	7.82
25	6.17	8.03
26	6.34	8.25
27	6.51	8.47
28	6.68	8.69
29	6.85	8.92
30	7.03	9.15
31	7.21	9.38
32	7.39	9.62
33	7.57	9.86
34	7.76	10.1
35	7.95	10.3

Table S5. DGT-measured solution concentrations (c_{DGT}), bulk solution concentrations (c_{soln}), and c_{DGT}/c_{soln} ratios of Pb in synthetic soil solutions [a]

		Pb concentration		
Solution	c(Ca) / mg L^{-1}	c_{DGT} / µg L^{-1}	c_{soln} / µg L^{-1}	c_{DGT}/c_{soln}
A	51.4 ± 1.6	13.3 ± 1.6	13.7 ± 0.3	0.97 ± 0.12
B	112 ± 10	16.5 ± 2.0	15.9 ± 0.7	1.04 ± 0.13
C	159 ± 6	15.6 ± 3.6	15.2 ± 0.9	0.93 ± 0.24

[a] Errors are expanded uncertainties (U, k = 2)

Table S6. Efficiency of TK100 DGT for separation of Sr and Pb from different potentially interfering matrix cations [a]

	Sr	Pb
Matrix cation	Matrix separation	
Mg^{2+}	99 ± 1 %	100 ± <1 %
K^{+}	93 ± <1 %	100 ± <1 %
Ca^{2+}	94 ± 2 %	100 ± <1 %
Rb^{+}	99 ± <1 %	100 ± <1 %

[a] Matrix separation is expressed as average relative difference between the elemental mass concentration ratio in the synthetic soil solutions and in the DGT eluates. Errors are 1*SD* (*n* = 3).

Table S7. Elemental mass concentrations in soil solutions of the experimental soils obtained by centrifugation of incubated soil pastes [a]

	c(Mg)	*c*(K)	*c*(Ca)	*c*(Rb)	*c*(Sr)	*c*(Pb)
Soil sample	mg L^{-1}	mg L^{-1}	mg L^{-1}	µg L^{-1}	µg L^{-1}	µg L^{-1}
SL	9.02 ± 1.70	6.90 ± 0.80	53.4 ± 9.41	13.3 ± 1.6	183 ± 36	0.95 ± 0.12
AC	17.7 ± 4.8	20.1 ± 3.0	59.6 ± 17.9	26.4 ± 3.0	218 ± 63	362 ± 23
MC	63.5 ± 2.6	5.43 ± 0.38	488 ± 23	13.9 ± 0.7	865 ± 30	108 ± 5

[a] Errors are expanded uncertainties (U, $k = 2$).

Table S8. Isotope ratios of Sr and Pb in NH_4NO_3 (c = 1 mol L^{-1}) extracts of the experimental soils [a]

Soil sample	$\delta_{SRM\ 987}(^{87}Sr/^{86}Sr)_{NH4NO3}$ / ‰	$\delta_{SRM\ 981}(^{207}Pb/^{206}Pb)_{NH4NO3}$ / ‰
SL	12.8 ± 0.3	n.m.
AC	0.33 ± 0.28	-67.2 ± 0.4
MC	-1.24 ± 0.29	-67.4 ± 0.3

[a] Errors are expanded uncertainties (U, k = 2). n.m. = not measured.

References

1. Kreuzeder, A.; Santner, J.; Zhang, H.; Prohaska, T.; Wenzel, W. W., Uncertainty evaluation of the diffusive gradients in thin films technique. *Environ. Sci. Technol.* **2015,** *49* (3), 1594-602.
2. Horsky, M.; Irrgeher, J.; Prohaska, T., Evaluation strategies and uncertainty calculation of isotope amount ratios measured by MC ICP-MS on the example of Sr. *Anal. Bioanal. Chem.* **2016,** *408* (2), 351-367.
3. Zhang, H.; Davison, W., Diffusional characteristics of hydrogels used in DGT and DET techniques. *Anal. Chim. Acta* **1999,** *398* (2), 329-340.
4. Scally, S.; Davison, W.; Zhang, H., Diffusion coefficients of metals and metal complexes in hydrogels used in diffusive gradients in thin films. *Anal. Chim. Acta* **2006,** *558* (1), 222-229.
5. Warnken, K. W.; Zhang, H.; Davison, W., Performance characteristics of suspended particulate reagent-iminodiacetate as a binding agent for diffusive gradients in thin films. *Anal. Chim. Acta* **2004,** *508* (1), 41-51.
6. Amelung, W.; Blume, H.-P.; Fleige, H.; Horn, R.; Kandeler, E.; Kögel-Knabner, I.; Kretzschmar, R.; Stahr, K.; Wilke, B.-M., *Scheffer/Schachtschabel Lehrbuch der Bodenkunde*. 17 ed.; Springer Spektrum: 2018.
7. Tyler, G.; Olsson, T., Plant uptake of major and minor mineral elements as influenced by soil acidity and liming. *Plant and Soil* **2001,** *230* (2), 307-321.
8. Kot, F. S., On the rubidium and lithium content and availability in the sub-arid south-eastern Mediterranean: potential health implications. *Environmental Geochemistry and Health* **2018,** *40* (5), 1841-1851.
9. Drouet, T.; Herbauts, J.; Gruber, W.; Demaiffe, D., Natural strontium isotope composition as a tracer of weathering patterns and of exchangeable calcium sources in acid leached soils developed on loess of central Belgium. *European Journal of Soil Science* **2007,** *58* (1), 302-319.
10. Takeda, A.; Tsukada, H.; Takaku, Y.; Akata, N.; Hisamatsu, S. i., Plant induced changes in concentrations of caesium, strontium and uranium in soil solution with reference to major ions and dissolved organic matter. *J. Environ. Radioact.* **2008,** *99* (6), 900-911.
11. Pett-Ridge, J. C.; Derry, L. A.; Barrows, J. K., Ca/Sr and 87Sr/86Sr ratios as tracers of Ca and Sr cycling in the Rio Icacos watershed, Luquillo Mountains, Puerto Rico. *Chem. Geol.* **2009,** *267* (1), 32-45.
12. Kabata-Pendias, A., *Trace Elements in Soils and Plants*. 4 ed.; Taylor & Francis Group: U.S., 2011.
13. Alva, A. K.; Sumner, M. E.; Miller, W. P., Relationship between ionic strength and electrical conductivity for soil solutions. *Soil Science* **1991,** *152* (4), 239-242.
14. Ernstberger, H.; Zhang, H.; Tye, A.; Young, S.; Davison, W., Desorption Kinetics of Cd, Zn, and Ni Measured in Soils by DGT. *Environ. Sci. Technol.* **2005,** *39* (6), 1591-1597.
15. DIN ISO 19730 Bodenbeschaffenheit - Extraktion von Spurenelementen aus Böden mit Ammoniumnitratlösung (ISO 19730:2008). https://shop.austrian-standards.at/action/de/public/details/338432/DIN_ISO_19730_2009_07 (accessed 08.11.2021).
16. Zimmermann, T.; Retzmann, A.; Schober, M.; Pröfrock, D.; Prohaska, T.; Irrgeher, J., Matrix separation of Sr and Pb for isotopic ratio analysis of Ca-rich samples via an automated simultaneous separation procedure. *Spectrochim. Acta, Part B* **2018**.
17. DGT Research Diffusion Coefficients. https://www.dgtresearch.com/diffusion-coefficients/ (accessed 29.01.2020).
